# Efficacy and safety of interventions for infantile hemangioma compared with oral propranolol: an updated systematic review and bayesian network meta-analysis

**DOI:** 10.1007/s00431-026-07257-y

**Published:** 2026-07-21

**Authors:** Caihong Li, Liehao Yang, Lingfeng Pan

**Affiliations:** 1 Department of Dermatology, University Hospital Erlangen, Friedrich-Alexander University of Erlangen-Nürnberg, 91054 Erlangen, Germany; 2 Department of Dermatology, China-Japan Union Hospital of Jilin University, Changchun City, 130033 Jilin Province China; 3 Department of Plastic and Hand Surgery, Klinikum Rechts Der Isar, School of Medicine, Technical University of Munich, 81675 Munich, Germany

**Keywords:** Infantile hemangioma, Therapy, Network meta-analysis, Propranolol, Atenolol, Treatment outcome, Drug safety

## Abstract

The purpose of the study is to compare the efficacy and safety of available interventions for infantile hemangioma against oral propranolol and to evaluate the certainty of the comparative evidence. We conducted a Bayesian network meta-analysis in accordance with PRISMA-NMA guidelines and systematically searched PubMed, Embase, the Cochrane Library, and CNKI from January 2008 to June 2026. A random-effects consistency model was fitted using the BUGSnet package. Evidence certainty was assessed using the CINeMA framework, and risk of bias was evaluated using the revised Cochrane RoB 2 tool. Thirty randomized controlled trials (RCTs) including 2,639 patients across nine treatment nodes were included in the efficacy analysis, and 12 RCTs involving 1,143 patients across eight nodes were included in the safety analysis. Using oral propranolol as the reference treatment, no active intervention demonstrated statistically significant superiority in efficacy, whereas placebo was significantly inferior. Corticosteroids were the only intervention associated with substantially higher adverse event rates, whereas atenolol showed a trend toward fewer adverse events. By anchoring all comparisons to oral propranolol and integrating CINeMA certainty assessments with inconsistency testing, these findings are consistent with the continued role of oral propranolol as the reference systemic treatment for infantile hemangioma. For patients intolerant to propranolol, atenolol may represent a reasonable alternative.

*Conclusions*: By anchoring all comparisons to oral propranolol and integrating CINeMA certainty assessments with inconsistency testing, these findings are consistent with the continued role of oral propranolol as the reference systemic treatment for infantile hemangioma. For patients intolerant to propranolol, atenolol may represent a reasonable alternative.

**Table:** 

**What is Known:** • *Oral propranolol is the established first-line systemic therapy for infantile hemangioma.* • *Previous network meta-analyses have reported treatment rankings, but the clinical meaning of these rankings relative to oral propranolol remains uncertain.*	
**What is New:** • *Despite favorable point estimates for combination therapy and nadolol, this benchmark-anchored analysis found no included active intervention statistically superior to oral propranolol.* • *Certainty assessment showed that treatment rankings were limited by heterogeneity, inconsistency, risk of bias, and imprecision; atenolol had the largest body of direct comparative evidence among the alternatives.*

**Supplementary Information:**

The online version contains supplementary material available at 10.1007/s00431-026-07257-y.

## Introduction

Infantile hemangioma (IH) is the most common benign vascular tumor of infancy, affecting approximately 4–5% of newborns, predominantly premature, low-birth-weight, and Caucasian females [1]. IH follows a characteristic triphasic natural history comprising a rapid proliferative phase during the first several months of life, a slow involuting phase beginning around 12 months of age, and an involuted phase in which residual fibrofatty tissue or telangiectasia may persist [2]. Although the majority of IH undergo spontaneous regression, approximately 10–15% require intervention because of ulceration, functional impairment (e.g., periorbital IH causing amblyopia, subglottic airway obstruction), disfigurement, or an association with extracutaneous anomalies such as PHACE or LUMBAR syndrome [3].

The treatment landscape for IH was transformed by the discovery of propranolol’s anti-angiogenic properties [4], establishing it as the first-line systemic therapy recommended by current clinical guidelines [5, 6]. Despite its dominance, concerns regarding propranolol’s adverse effects—including sleep disturbances, bronchospasm, hypoglycemia, and bradycardia—have prompted the search for alternative treatments [7]. Consequently, alternative interventions—including β_1_-selective blockers (e.g., atenolol) [8], other non-selective β-blockers (e.g., nadolol) [9], topical therapies [10], and combination strategies [11]—have emerged to optimize the efficacy-to-safety ratio, while historical mainstays like corticosteroids have been largely supplanted [12].

Previous network meta-analyses (NMAs) have laid a valuable foundation for understanding IH treatment outcomes. Early syntheses effectively evaluated core pharmacological interventions [13] and preliminary treatment hierarchies [14]. As the therapeutic landscape expands, there is now an opportunity to build upon these large-scale networks by incorporating newer beta-blockers and physical therapies, alongside applying advanced methodological frameworks like formal certainty of evidence evaluations [15]. Furthermore, while previous syntheses, including Cochrane reviews [16], have informatively framed the clinical question around absolute probability rankings (e.g., SUCRA), the evolving clinical paradigm now necessitates a more targeted inquiry. Rather than focusing solely on overall rankings, it is increasingly relevant to ask: does any available alternative demonstrate statistical superiority over oral propranolol, the established standard of care?

To address this gap, we conducted an updated Bayesian NMA to systematically compare the efficacy and safety of available interventions for IH. We anchored our analysis against oral propranolol as the explicit benchmark to evaluate whether any alternative demonstrates statistical superiority in efficacy or a more favorable safety profile. Furthermore, by applying the Confidence in Network Meta-Analysis (CINeMA) framework and performing inconsistency testing, we aim to provide a comprehensive evidence synthesis to inform clinical decision-making.

## Materials and methods

### Protocol and registration

We conducted a systematic review and Bayesian network meta-analysis (NMA) to assess the efficacy and safety of available interventions for infantile hemangioma (IH). This study adhered to the Preferred Reporting Items for Systematic Reviews and Meta-Analyses extension for Network Meta-Analyses (PRISMA-NMA) guidelines. The protocol was prospectively registered in the International Prospective Register of Systematic Reviews (PROSPERO; Registration No.: CRD420261286410).

### Search strategy and study selection

We systematically searched PubMed, Embase, the Cochrane Library, and CNKI from January 2008 (the year propranolol was introduced for infantile hemangioma) to June 8, 2026. The search strategy combined controlled vocabulary and terms related to infantile hemangioma, therapeutic interventions (e.g., beta-blockers, corticosteroids, laser therapy), and randomized trial designs. We included studies if they (i) were randomized controlled trials (RCTs); (ii) enrolled infants or children clinically diagnosed with infantile hemangioma; and (iii) compared at least two interventions (including placebo or observation) with extractable efficacy or safety data. Conversely, we excluded studies if they (i) utilized non-randomized designs; (ii) lacked sufficient quantitative data; (iii) investigated other vascular anomalies (e.g., congenital hemangiomas or vascular malformations) without separable data for infantile hemangioma; or (iv) were duplicate publications. For overlapping cohorts, we retained the most recent and comprehensive dataset.

### Data extraction

Two independent reviewers sequentially screened titles, abstracts, and full texts, with a third reviewer resolving any discrepancies. Data were extracted using a standardized, piloted electronic form capturing study characteristics (first author, publication year, country, design, and blinding); demographics (sample size, age at enrollment, IH type, and location); and intervention specifics (regimen, dosage, route, laser parameters, and duration). Intention-to-treat (ITT) data were prioritized for all analyses. The primary efficacy outcome, treatment success rate, was defined as complete lesion resolution or a > 75% reduction in size at the assessment endpoint. The primary safety outcome was the proportion of patients experiencing treatment-related adverse events (e.g., ulceration, hypotension, sleep disturbances, or gastrointestinal symptoms). To ensure temporal consistency across the network, we prioritized outcomes assessed at 6 months post-initiation; if unavailable, the closest reported time point was used. Treatment interventions were classified into network nodes based on pharmacological class and route of administration. Oral beta-blockers were separated by selectivity profile into propranolol (non-selective), atenolol (β1-selective), and nadolol (non-selective, longer-acting). Topical beta-blockers, including timolol maleate and carteolol hydrochloride, were grouped into a single node given their shared non-selective β-adrenergic mechanism and identical topical route. Laser therapies, including pulsed dye laser and Nd:YAG laser, were grouped as a single node because both represent photothermal interventions and the limited number of studies per modality precluded separate analysis. Propranolol combination therapy was defined as oral propranolol administered concurrently with any adjunctive modality; this pragmatic grouping was necessary to maintain network connectivity, as each specific combination was evaluated in only one or two trials. The heterogeneity within this node is acknowledged as a limitation and examined through inconsistency testing. Study authors were not contacted for additional data, and no imputation was performed. Studies not reporting adverse event data were excluded from the safety analysis; zero-event cells were handled within the Bayesian framework without continuity corrections.

### Risk of bias and certainty of evidence

We assessed the risk of bias in all included RCTs using the revised Cochrane risk-of-bias tool (RoB 2). Two independent reviewers evaluated the five standard domains (randomization process, deviations from intended interventions, missing outcome data, outcome measurement, and selection of the reported result) to assign an overall risk of low, some concerns, or high. Evidence certainty for key network estimates was evaluated using the Confidence in Network Meta-Analysis (CINeMA) framework, which adapts the GRADE approach for network meta-analyses [17]. Overall certainty was rated as high, moderate, low, or very low based on six domains: within-study bias, reporting bias, indirectness, imprecision, heterogeneity, and incoherence.

### Statistical analysis

All analyses were performed using R version 4.3.2 (R Foundation for Statistical Computing, Vienna, Austria). We used the *netmeta* package (version 2.9–0) to visualize network geometry, with node sizes proportional to the number of randomized patients and edge thicknesses reflecting the number of direct comparisons.

The primary Bayesian NMA was conducted using the *BUGSnet* package interfacing with JAGS (Just Another Gibbs Sampler). Dichotomous outcomes were modeled using a binomial likelihood with a logit link function. We fitted a consistency random-effects model to account for between-study heterogeneity, applying non-informative priors: Normal (0, 100^2^) for relative treatment effects and baselines, and a Uniform (0, 2) prior for the heterogeneity parameter (τ). Posterior distributions were estimated via Markov Chain Monte Carlo (MCMC) simulations using three parallel chains of 50,000 iterations following a 20,000-iteration burn-in. Convergence was confirmed using trace plots and the Gelman–Rubin diagnostic (*R̂* < 1.05).

Local inconsistency was evaluated using the node-splitting method, while global inconsistency was assessed by comparing the deviance information criterion (DIC) between consistency and inconsistency models, alongside the design-by-treatment interaction Q-statistic from *netmeta*. Potential small-study effects and publication bias were explored using comparison-adjusted funnel plots and Egger-type tests. Relative treatment effects are reported as posterior median odds ratios (ORs) with 95% credible intervals (CrIs). Treatment rankings were summarized using rankograms and the surface under the cumulative ranking curve (SUCRA), where higher values indicated greater efficacy or a lower incidence of adverse events (better safety). We conducted a sensitivity analysis excluding studies judged to be at high risk of bias to assess the robustness of the primary network estimates.

## Results

### Study selection and characteristics

**Fig. 1:**
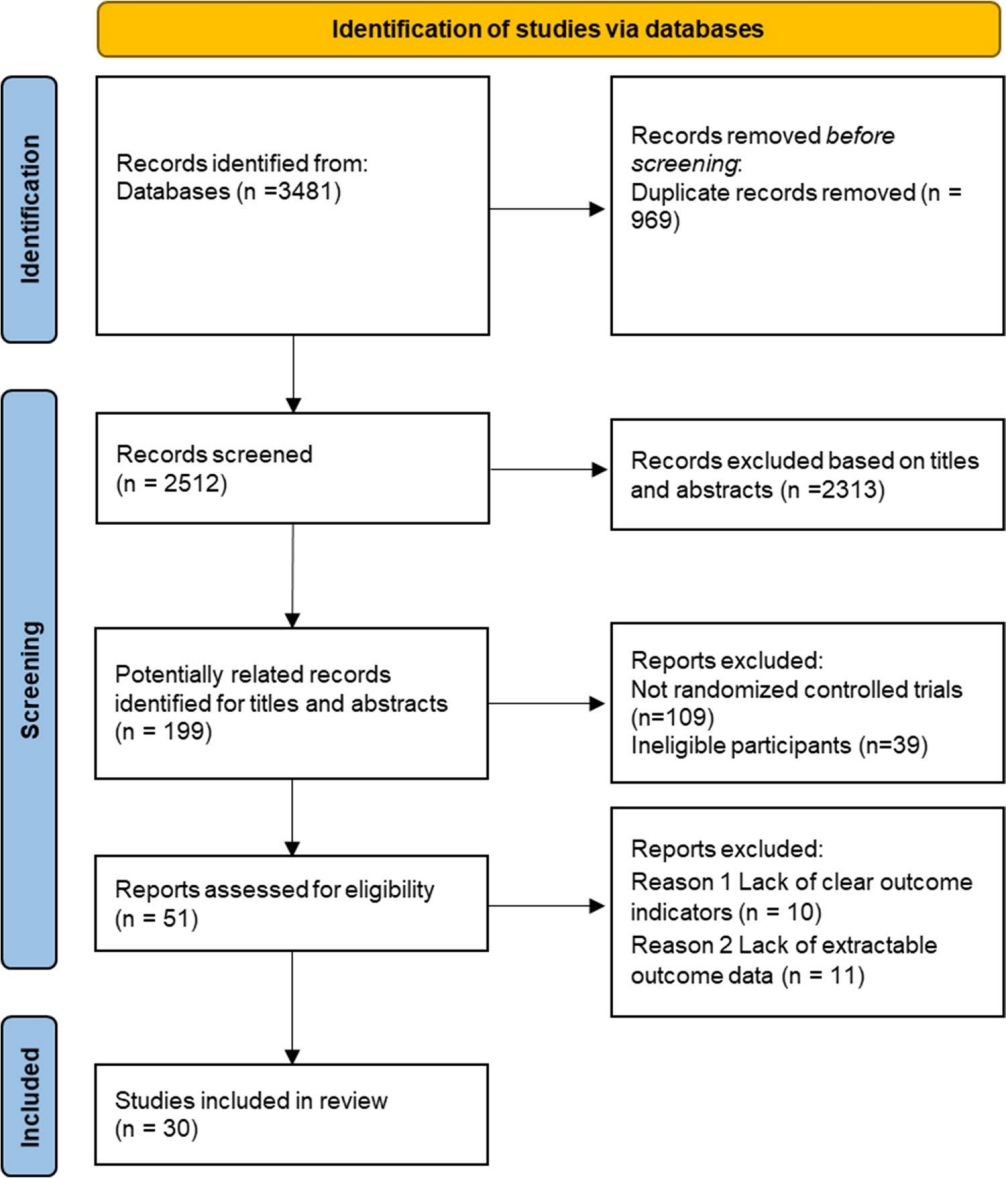
PRISMA flow diagram of the study selection process. A total of 3481 records were identified, and 30 randomized controlled trials were included in the final network meta-analysis. PRISMA, Preferred Reporting Items for Systematic Reviews and Meta-Analyses

Our systematic search yielded 3481 records from PubMed, Embase, the Cochrane Library, and CNKI. After removing 969 duplicates, we screened the titles and abstracts of the remaining 2512 records, identifying 199 potentially relevant articles for full-text review. Following full-text assessment, 148 records were excluded (109 non-RCTs and 39 enrolling ineligible participants). Of the remaining 51 reports, a further 21 were excluded due to unclear outcome indicators (*n* = 10) or unextractable data (*n* = 11). Ultimately, 30 RCTs reporting efficacy and 12 reporting safety outcomes were included in the network meta-analysis. This selection process is detailed in the PRISMA flow diagram (Fig. 1).

The 30 efficacy trials encompassed 2639 patients across 9 treatment nodes: oral propranolol, atenolol, nadolol, intralesional propranolol, topical beta-blockers (e.g., timolol maleate, carteolol), laser therapy (pulsed dye laser (PDL) or Nd:YAG), corticosteroids (systemic or topical), propranolol combination therapy (oral propranolol plus another modality, such as laser or topical timolol), and placebo/observation. The 12 safety trials involved 1143 patients across 8 treatment nodes. Included trials were published between 2013 and 2025 across 11 countries, predominantly in China (*n* = 10). Study designs included 9 double-blind, 10 single-blind, and 11 open-label RCTs. Detailed trial characteristics are summarized in Table 1 and Supplementary Table S1.

**Table 1:** Characteristics of included studies

Study (first author, year)	Country	Study design (blinding)	Total *N*	Age at enrollment	IH type/location	Comparisons	Assessment timepoint	Outcomes reported	RoB 2 judgment
Gan LQ 2018	China	Single-blind RCT	349	3.5 (1.2–12.0)	Superficial IHs	Topical beta-blockers vs placebo	3 mo	Efficacy	High
Christine Léauté-Labrèze 2015	France	Double-blind RCT	156	1 to 5 months	Proliferating infantile hemangioma	Propranolol vs placebo	6 mo	Efficacy, AE	Low
M Dakoutrou 2019	Greece	Single-blind RCT	54	3.63 ± 1.31	Superficial IHs/facial and/or neck	Atenolol vs propranolol	6 mo	Efficacy, AE	High
Alvaro Ábarzúa-Araya 2014	Chile	Double-blind RCT	23	1 to 15 months 5.2 ± 3.5 months	Superficial and mixed IHs	Atenolol vs propranolol	6 mo	Efficacy	Low
Amir Hooshang Ehsani 2014	Iran	Open-label RCT	19	3–13 months	Superficial	Propranolol combination vs laser	3 mo	Efficacy	High
Hatem M Marey 2017	Egypt	Single-blind RCT	25	1 to 6 months	Early proliferative superficial periocular infantile capillary hemangioma	Propranolol combination vs propranolol	6 mo	Efficacy	Some concerns
Ashraf, Raihan 2023	India	Single-blind RCT	55	5.2 ± 2.88	IHs	Atenolol vs propranolol	9 mo	Efficacy	Low
Fan Ma 2024	China	Open-label RCT	67	1.1–7.2 months	Face, trunk, and limbs IHs	Propranolol combination vs propranolol	6 mo	Efficacy	High
QY Chen 2021	China	Open-label RCT	19	2–11months	IHs complicated with recently formed ulcers	Topical beta-blockers vs laser	1 mo	Efficacy	High
Yi Ji 2021	China	Double-blind RCT	377	5 and 20 weeks	Problematic IHs who required systemic therapy	Atenolol vs propranolol	6 mo	Efficacy	Low
Fania Z Muñoz-Garza 2021	Spain	Double-blind RCT	50	10 to 60 days	Focal or segmental hemangiomas	Topical beta-blockers vs placebo	6 mo	Efficacy, AE	Low
Sunita Singh 2025	India	Double-blind RCT	40	Less than 24 months	Superficial to deep dermal IHs	Topical beta-blockers vs steroid	6 mo	Efficacy	Some concerns
He Gong 2015	China	Open-label RCT	39	2–9 months	Superficial haemangiomas	Propranolol combination vs propranolol vs topical beta-blockers	6 mo	Efficacy	High
E. Pope 2013	Canada	Double-blind RCT	19	1–12 months	Head and neck IHs	Nadolol vs propranolol	6 mo	Efficacy	Low
Elena Pope 2022	Canada	Double-blind RCT	71	1 to 6 months	Hemangioma greater than 1.5 cm on the face or 3 cm or greater on another body part	Nadolol vs propranolol	6 mo	Efficacy, AE	Low
Yanyan Guo 2025	China	Open-label RCT	260	3–11 months	IHs	Propranolol vs propranolol combination	6 mo	Efficacy, AE	High
Hesham Zaher 2025	Egypt	Single-blind RCT	45	Less than 24months	IHs	Propranolol vs topical beta-blockers vs intralesional propranolol	6 mo	Efficacy	High
Nancy M Bauman 2014	USA	Double-blind RCT	19	2 weeks and 6 months	Proliferating IH	Propranolol vs steroid	4 mo	Efficacy, AE	Low
Preeti Tiwari 2016	India	Single-blind RCT	64	4.61 ± 1.10 months	Ulcerated haemangioma of head and neck	Propranolol vs placebo	6 mo	Efficacy, AE	High
Xinjun Sun 2018	China	Open-label RCT	100	1–7 months	Single hemangiomas	Propranolol combination vs laser	6 mo	Efficacy, AE	High
Janneke P H M Kessels 2013	Netherlands	Single-blind RCT	19	1.5 to 5 months	Superficial hemangioma	Laser vs placebo	Followed up until the age of 1 year	Efficacy	High
Mohamed M D Aly 2015	Egypt	Single-blind RCT	40	4 weeks–8 months	Cutaneous hemangioma	Propranolol combination vs propranolol	6 mo	Efficacy, AE	High
Aditi Mehta 2019	India	Single-blind RCT	20	8–20 months	Periorbital and eyelid capillary hemangiomas	Intralesional propranolol vs propranolol	6 mo	Efficacy	High
Retno Danarti 2016	Indonesia	Single-blind RCT	185	1–11 months	Superficial IH	Topical beta-blockers vs steroid	6 mo	Efficacy	Some concerns
Abeer A Tawfik 2015	Egypt	Open-label RCT	60	13.4 ± 9.4 months	IHs	Topical beta-blockers vs laser	6 mo	Efficacy	High
Tao Wang 2024	China	Open-label RCT	33	1–6 months	IHs	Atenolol vs propranolol	6 mo	Efficacy, AE	Some concerns
Shuxia Zhong 2015	China	Open-label RCT	60	1 to 12 months	Mixed and deep IHs	Propranolol vs laser vs propranolol combination	6 mo	Efficacy	High
Wang Qi 2016	China	Open-label RCT	75	5–20 weeks	Superficial or mixed IH	Atenolol vs propranolol	6 mo	Efficacy	High
Guanjie Wang 2016	China	Single-blind RCT	123	1 to 14 months	IHs	Atenolol vs propranolol	6 mo	Efficacy, AE	Some concerns
Longlong Sun 2018	China	Single-blind RCT	173	1–5 months	Proliferating IH	Atenolol vs propranolol	6 mo	Efficacy, AE	High

### Network geometry

**Fig. 2:**
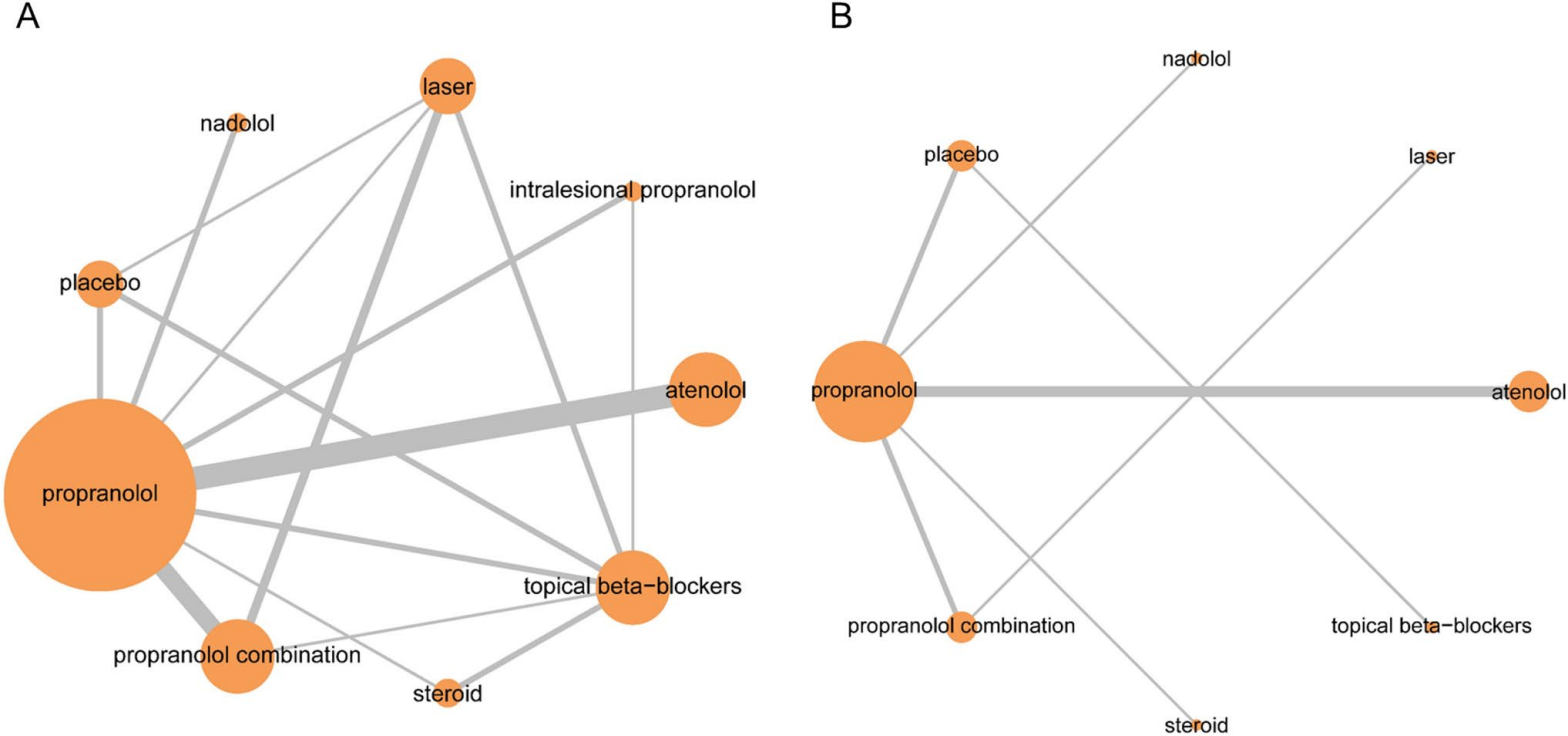
Network plots for the efficacy (**A**) and safety (**B**) outcomes. Node size is proportional to the number of patients, and edge thickness is proportional to the number of studies. The efficacy network comprised 9 nodes and 63 study arms from 30 RCTs; the safety network comprised 8 nodes and 24 study arms from 12 RCTs

Figure 2 presents the network geometry for both outcomes. The efficacy network (Fig. 2A) comprised 30 RCTs (63 study arms) across 9 nodes, featuring 27 two-arm and three three-arm trials [18–47]. Oral propranolol served as the central hub in this well-connected network, which lacked any disconnected subnetworks. Similarly, the safety network (Fig. 2B) involved 12 RCTs (24 study arms) across 8 nodes, again centered on oral propranolol.

### Risk of bias assessment

The risk of bias assessments (RoB 2) are summarized in Supplementary Figures S1 and S2. Overall, 8 trials (26.7%) exhibited a low risk of bias, 5 (16.7%) raised some concerns, and 17 (56.7%) were classified as high risk. Across the five domains, outcome measurement (D4) and deviations from intended interventions (D2) were the most frequent sources of bias. Specifically, 19 trials (63.3%) carried some concerns or high risk in D4, primarily due to subjective clinical evaluations (e.g., visual analog scales or photographs) and unblinded assessors. Similarly, 15 trials (50.0%) showed concerns or high risk in D2, largely driven by open-label or single-blind designs. Conversely, missing outcome data (D3) posed the least concern, with 28 trials (93.3%) rated as low risk. The randomization process (D1) and selection of the reported result (D5) also performed relatively well, with 23 (76.7%) and 20 (66.7%) trials achieving a low-risk rating, respectively.

### Model selection and convergence assessment

For the primary efficacy outcome, the random-effects consistency model provided the optimal fit, yielding a substantially lower residual deviance (*D*_*res*_ = 58.48) than the fixed-effects model. This *D*_*res*_ closely matched the 63 data points, indicating adequate model fit. The consistency assumption was supported by a lower deviance information criterion (DIC) compared to the inconsistency model (58.64 vs. 59.91). Convergence across all MCMC chains was confirmed via trace plots and the Gelman–Rubin diagnostic (all *R̂* < 1.002). Conversely, a fixed-effects consistency model was more appropriate for the safety outcome, yielding a lower DIC (42.58). The model’s *D*_*res*_ (22.67) aligned with the 24 study arms, and nearly identical DIC values between consistency and inconsistency models indicated no evidence of inconsistency. Convergence was similarly achieved for this network (all *R̂* < 1.005).

### Efficacy: pairwise comparisons

**Fig. 3:**
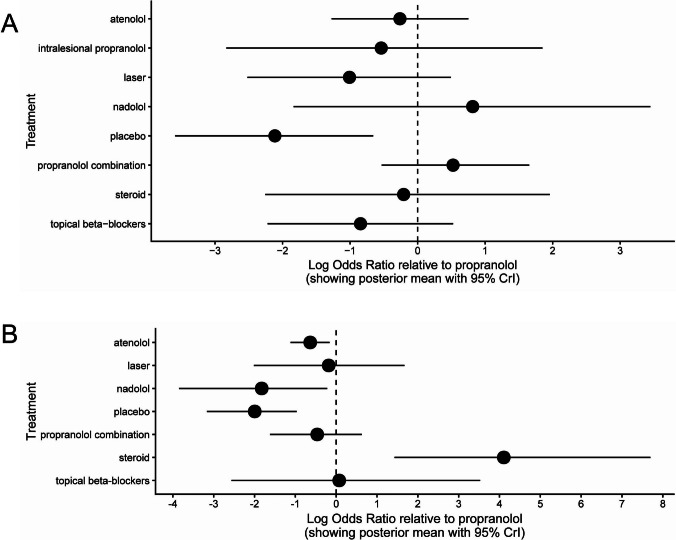
Forest plots of the Bayesian network meta-analysis results versus oral propranolol (reference) for efficacy (**A**) and safety (**B**) outcomes. Point estimates represent posterior mean log odds ratios with 95% credible intervals. The dashed line indicates no difference relative to propranolol. OR, odds ratio; CrI, credible interval

The league table of all pairwise comparisons and forest plots for treatment success rate are presented in Table 2 and Fig. 3A. Using oral propranolol as the reference, only placebo/observation showed a statistically significant difference (OR = 0.12, 95% CrI: 0.03–0.52), demonstrating propranolol’s superiority over no active treatment. None of the remaining seven interventions differed significantly from propranolol: atenolol (OR = 0.77, 95% CrI: 0.28–2.13), corticosteroids (OR = 0.80, 95% CrI: 0.11–7.24), propranolol combination therapy (OR = 1.67, 95% CrI: 0.59–5.21), intralesional propranolol (OR = 0.58, 95% CrI: 0.06–6.33), topical beta-blockers (OR = 0.43, 95% CrI: 0.11–1.70), laser therapy (OR = 0.36, 95% CrI: 0.08–1.62), and nadolol (OR = 2.27, 95% CrI: 0.16–33.70). SUCRA values are reported in Table 2 and Supplementary Figure S4 for reference.

**Table 2:**
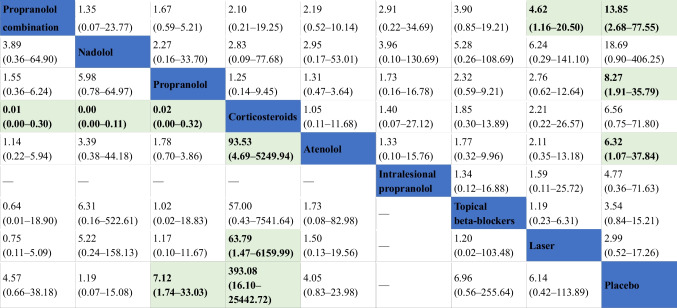
Combined league table for efficacy and safety outcomes

Upper triangle (read left to right): efficacy (treatment success rate), expressed as OR (95% CrI); an OR > 1 favors the row treatment over the column treatment. Lower triangle (read left to right): safety (adverse events), expressed as OR (95% CrI); an OR > 1 indicates a higher likelihood of adverse events for the column treatment compared with the row treatment. Statistically significant results (95% CrI excluding 1) are shown in bold with green shading. Treatments on the diagonal are ordered by SUCRA ranking for efficacy (highest to lowest). The em dash “—" indicates comparisons unavailable in the safety network (intralesional propranolol was not included in the adverse event analysis). *OR* odds ratio, *CrI* credible interval, *SUCRA* surface under the cumulative ranking curve

### Safety: adverse event comparisons

The NMA results for adverse events are presented in Table 2 and Fig. 3B, where an OR greater than 1 indicates higher adverse event likelihood relative to the reference. Compared with oral propranolol, corticosteroids were associated with higher odds of adverse events (OR = 52.92, 95% CrI: 3.12–2,874.40), whereas placebo showed significantly lower odds (OR = 0.14, 95% CrI: 0.03–0.57). No other treatment differed significantly from propranolol. Atenolol showed a non-significant trend toward fewer adverse events (OR = 0.56, 95% CrI: 0.26–1.43).

### Heterogeneity and inconsistency assessments

Substantial between-study heterogeneity was observed in the efficacy network (τ = 1.18). Global inconsistency testing was significant, but node-splitting localized this to a single source: conflicting direct and indirect evidence for the propranolol versus combination therapy comparison. The remaining 12 comparisons showed no significant inconsistency (Supplementary Table S2). Frequentist estimates from the netmeta package were consistent with the primary Bayesian results (Supplementary Table S3). The safety network exhibited negligible heterogeneity and no inconsistency, supporting the use of a fixed-effects model.

### Publication bias assessment

Neither the comparison-adjusted funnel plots (Supplementary Figure S3) nor the Egger regression tests revealed significant asymmetry for either the efficacy (*P* = 0.12) or safety (*P* = 0.08) outcomes, suggesting no strong evidence of publication bias or small-study effects. Nevertheless, these findings should be interpreted cautiously given the limited number of studies per comparison, particularly in the safety network.

### Certainty of evidence

Evidence certainty for key comparisons against oral propranolol (CINeMA framework) ranged from moderate to very low (Supplementary Table S4). Placebo versus oral propranolol yielded the highest certainty (moderate), providing evidence of propranolol’s superiority despite minor downgrades for within-study bias and heterogeneity. Atenolol versus propranolol, despite offering the largest body of direct evidence, was graded as low certainty due to concerns regarding bias, imprecision, heterogeneity, and incoherence. All remaining comparisons—including combination therapy, intralesional propranolol, corticosteroids, laser therapy, topical beta-blockers, and nadolol—were rated as very low certainty. These were primarily downgraded due to major concerns with imprecision (often driven by limited sample sizes or reliance on indirect evidence) and within-study bias, alongside varying degrees of heterogeneity and incoherence.

### Sensitivity analysis

After excluding studies judged to be at high risk of bias, 13 trials remained, forming a connected seven-node network. Laser therapy and intralesional propranolol were absent because all trials contributing to these nodes were judged to be at high risk of bias. The core findings were consistent with the primary analysis: no active treatment demonstrated significant superiority over oral propranolol, and placebo remained significantly inferior (OR = 0.04). The direction and magnitude of treatment effects were broadly stable across comparisons (Supplementary Table S5). Notably, the consistency and inconsistency models yielded nearly identical residual deviance in this restricted network, suggesting that the inconsistency observed in the primary analysis was largely attributable to high risk-of-bias studies.

## Discussion

In this updated Bayesian network meta-analysis encompassing 30 RCTs and 2639 patients, we systematically evaluated nine interventions for infantile hemangioma. The main finding of this analysis is that no active intervention showed statistically significant superiority over oral propranolol in treatment success. Placebo was significantly inferior, consistent with propranolol’s established therapeutic benefit; all other active interventions yielded comparable efficacy. Regarding safety, corticosteroids emerged as the sole intervention associated with a significantly higher risk of adverse events compared to propranolol, whereas the remaining active treatments exhibited comparable or potentially more favorable safety profiles. Collectively, these findings support oral propranolol’s continued role as the reference treatment for IH management, while providing a comparative framework to inform individualized therapeutic decisions.

While our findings broadly align with prior NMAs, they offer a complementary clinical perspective. Previous syntheses by Chinnadurai et al. [13] and Yang et al. [14] consistently placed oral propranolol at the top of probability rankings, whereas Fei et al. [15] highlighted the potential of combination strategies albeit with considerable imprecision. However, these earlier analyses primarily focused on identifying the highest-ranked treatment, rather than evaluating whether any alternative is statistically superior to the current standard of care. By explicitly anchoring all comparisons to propranolol, we found no evidence to support replacing it as the primary systemic therapy for IH. Ultimately, by synthesizing direct and indirect evidence across nine interventions within a unified Bayesian framework, our study provides a comprehensive statistical evaluation of this clinical paradigm.

Among the evaluated alternatives, atenolol appears to be a reasonable option when balancing efficacy and safety. Our synthesis suggests comparable efficacy to propranolol while exhibiting a distinct trend toward fewer adverse events. This finding is supported by the largest head-to-head trial by Ji et al. [27], alongside earlier comparative studies [21, 48], which consistently report similar overall response rates with a trend toward better tolerability. Although previous pairwise meta-analyses suggest propranolol might hold a slight edge in achieving complete clearance, this marginal benefit is frequently offset by a substantially higher burden of adverse events [49]. Pharmacologically, this favorable safety profile is plausible: atenolol’s β1-adrenoceptor selectivity circumvents β2-mediated complications (e.g., hypoglycemia and bronchospasm). Furthermore, its hydrophilicity limits blood–brain barrier penetration, markedly reducing sleep disturbances and central nervous system effects [50]. Consequently, atenolol may be considered a reasonable alternative for patients intolerant to propranolol or those with contraindications to non-selective β-blockade, such as reactive airway disease.

Nadolol yielded a favorable point estimate for efficacy (OR = 2.27, 95% CrI: 0.16–33.70), but this finding is based on only two small trials totaling 90 patients, and the extremely wide credible interval precludes meaningful clinical interpretation. Moreover, a reported fatal outcome linked to nadolol accumulation in an infant [51] raises serious pharmacovigilance concerns that cannot be addressed by the current evidence base. Therefore, nadolol requires adequately powered safety studies before it can be considered a viable alternative.

Although the point estimate favored propranolol combination therapy over propranolol monotherapy, the credible interval was wide and crossed the null (OR = 1.67, 95% CrI: 0.59–5.21). This node was also the primary source of both heterogeneity and inconsistency in our network (node-splitting *P* = 0.036). This instability likely reflects the clinical heterogeneity within this node, which grouped oral propranolol with diverse co-interventions including topical timolol, pulsed dye laser, Nd:YAG laser, intralesional lauromacrogol, bleomycin, and corticosteroids. The additional procedural burden associated with adjunctive laser or injection therapies—including repeated interventions, procedural discomfort, and increased costs—may not be justified in routine clinical practice. The role of specific combination regimens warrants evaluation in future trials designed to isolate the contribution of individual adjunctive components.

Regarding older systemic alternatives, corticosteroids were the only intervention associated with a substantially higher point estimate for adverse events (OR = 52.92, 95% CrI: 3.12–2,874.40), although the extremely wide credible interval reflects considerable imprecision driven by small sample sizes. A trend toward inferior efficacy was also observed. These findings are consistent with previous comparative trials [52, 53] and the known developmental toxicity of corticosteroids in infants, including growth retardation, cushingoid features, and adrenal suppression. Taken together, these results support current guideline recommendations that position systemic corticosteroids as second-line therapy for propranolol-refractory or contraindicated cases [5].

Topical beta-blockers and laser therapy, evaluated as standalone monotherapies, yielded lower treatment success rates compared with systemic propranolol. However, this difference likely reflects their distinct clinical indications rather than therapeutic inadequacy. This distinction is also relevant to the transitivity assumption: trials of topical beta-blockers and laser therapy generally enrolled superficial, localized, or ulcerated lesions, whereas systemic beta-blocker trials included a broader range of problematic, mixed, or deep IH requiring systemic therapy. These differences reflect clinical practice but may limit the validity of indirect comparisons between localized and systemic interventions, particularly because lesion depth, location, ulceration status, and age at treatment initiation were not consistently reported across treatment arms. As supported by recent trials [28, 54] and real-world evidence from Xia et al. [55], topical timolol or targeted laser therapy may remain appropriate for selected superficial or localized hemangiomas, especially when the clinical objective is localized control while avoiding systemic exposure.

Several methodological and clinical limitations constrain the certainty of our effect estimates. First, substantial heterogeneity was observed in the efficacy network (*τ* = 1.18), and global inconsistency was primarily related to the comparison involving propranolol combination therapy. This likely reflects the clinical diversity of the included trials and the pragmatic grouping of diverse regimens into a single “combination therapy” node to maintain network connectivity. In addition, several treatment nodes were supported by only a small number of trials, including nadolol, intralesional propranolol, corticosteroids, laser therapy, and specific combination regimens. This sparse evidence base contributed to wide credible intervals, greater reliance on indirect evidence, and unstable ranking probabilities.

Although oral propranolol served as the common reference treatment, propranolol regimens varied across trials. Most studies used 2.0–3.0 mg/kg/day, consistent with current guideline recommendations [5, 6], whereas four studies used 1.0–1.5 mg/kg/day (Supplementary Table S1), which may have underestimated the efficacy of the reference treatment and biased relative estimates in favor of comparators. Treatment duration was also variable: although most trials assessed outcomes at approximately 6 months, the remaining studies used time points ranging from 1 to 9 months. Patient age at enrollment generally fell within infancy, but several studies included infants beyond 12 months, when spontaneous involution may begin to confound treatment response. We did not perform dose-, duration-, or age-stratified subanalyses because the relevant subgroups were sparse and internally heterogeneous, and age was reported as study-level ranges rather than individual patient data; however, three of the four sub-guideline-dose studies were already excluded in the high risk-of-bias sensitivity analysis, which showed stable core findings.The primary efficacy outcome also involved measurement heterogeneity. To harmonize dichotomous efficacy data across trials, treatment success was defined as either complete lesion resolution or a > 75% reduction in lesion size. However, these endpoints are not clinically identical: complete resolution represents a stricter outcome, whereas a > 75% reduction may still include residual discoloration, fibrofatty tissue, or telangiectasia. In addition, included trials used different assessment methods, including clinical examination, photographic review, and investigator- or parent-rated scales. Such variability may have introduced outcome misclassification and contributed to between-study heterogeneity and imprecision in indirect comparisons.

A significant proportion of included trials (17/30, 56.7%) carried a high risk of bias, primarily driven by open-label designs and subjective outcome assessments, although maintaining strict double-blinding in IH trials is inherently challenging given that beta-blockers elicit visible physiological changes that can unmask treatment allocation. Additionally, the safety estimates should be interpreted with caution because all adverse events were pooled as a single composite outcome. Most trials reported only aggregate event counts and did not consistently classify events by type or severity, precluding event-specific analyses. This approach may obscure clinically important differences in safety profiles across interventions: systemic beta-blockers are mainly associated with cardiovascular, respiratory, metabolic, and sleep-related events; topical or laser therapies more often involve local irritation, pain, ulceration, or pigmentary changes; and corticosteroids raise concerns regarding growth and endocrine effects. Therefore, the safety network estimates compare overall adverse-event incidence rather than treatment-specific safety profiles or risks of specific clinically important adverse events. Finally, the safety network remains statistically underpowered to detect rare adverse events, and the literature lacks extended follow-up data addressing long-term rebound growth and neurodevelopmental outcomes.

To overcome current evidence gaps, future research should prioritize two critical areas. First, adequately powered, head-to-head non-inferiority trials—particularly comparing propranolol and atenolol—using standardized core outcome sets are urgently needed to resolve measurement heterogeneity. Second, given the ethical and logistical challenges of conducting large, blinded RCTs in infants, greater emphasis on real-world evidence (RWE) is warranted. Large-scale prospective registries and observational cohorts are essential to capture rare adverse events, evaluate the practical utility of combination therapies, and provide critical long-term data on relapse rates and neurodevelopmental outcomes following systemic beta-blocker exposure.

## Conclusion

This network meta-analysis suggests that no currently available intervention demonstrates statistically significant superiority over oral propranolol for infantile hemangioma. Systemic corticosteroids appear to have an unfavorable safety profile, while atenolol may provide a reasonable alternative for patients intolerant to propranolol. The clinical applicability of combination therapies and nadolol remains uncertain due to limited evidence. Overall, these findings support oral propranolol as the reference first-line treatment for infantile hemangioma.

## Supplementary Information

Below is the link to the electronic supplementary material.

ESM 1 Supplementary Material 1 (DOCX 13.3 MB)

ESM 2 Supplementary Material 2 (DOCX 14.0 KB)

ESM 3 Supplementary Material 3 (DOCX 38.1 KB)

## Data Availability

All relevant data has been provided in figures, tables and text.
