# Supplementary material for: Efficacy and safety of interventions for infantile hemangioma compared with oral propranolol: an updated systematic review and bayesian network meta-analysis
Source: Eur J Pediatr. 2026 Jul 21;185(8):595. doi: 10.1007/s00431-026-07257-y (PMC13388786; doi:10.1007/s00431-026-07257-y)
Supplement: Supplementary file 2 — Supplementary Material 2 (DOCX 14.0 KB) [file 431_2026_7257_MOESM2_ESM.docx]

Full search strategies

The complete reproducible search strategies for PubMed, Embase, and the Cochrane Central Register of Controlled Trials are presented below. CNKI was searched using combinations of Chinese-language terms corresponding to infantile hemangioma, therapeutic interventions, and randomized clinical trials. Because the CNKI search interface automatically processes and maps Chinese-language terms and does not generate a stable, directly reproducible search-string format, the search strategy is described narratively rather than presented as a verbatim search string.

PubMed:

(

"Hemangioma"[Mesh]

OR "infantile hemangioma"[Title/Abstract]

OR "infantile haemangioma"[Title/Abstract]

OR "hemangioma of infancy"[Title/Abstract]

OR "haemangioma of infancy"[Title/Abstract]

)

AND

(

"Adrenergic beta-Antagonists"[Mesh]

OR propranolol[Title/Abstract]

OR atenolol[Title/Abstract]

OR nadolol[Title/Abstract]

OR timolol[Title/Abstract]

OR carteolol[Title/Abstract]

OR beta-block*[Title/Abstract]

OR corticosteroid*[Title/Abstract]

OR steroid*[Title/Abstract]

OR prednisolone[Title/Abstract]

OR laser*[Title/Abstract]

OR bleomycin[Title/Abstract]

OR lauromacrogol[Title/Abstract]

OR therap*[Title/Abstract]

OR treat*[Title/Abstract]

OR intervention*[Title/Abstract]

)

AND

(

randomized controlled trial[Publication Type]

OR controlled clinical trial[Publication Type]

OR randomized[Title/Abstract]

OR randomised[Title/Abstract]

OR randomly[Title/Abstract]

OR placebo[Title/Abstract]

OR trial[Title/Abstract]

)

NOT (animals[MeSH Terms])

Cochrane

1. exp Hemangioma/

2. ("infantile hemangioma" or "infantile haemangioma" or

"hemangioma of infancy" or "haemangioma of infancy").ti,ab,kw.

3. exp Adrenergic beta-Antagonists/

4. (propranolol or atenolol or nadolol or timolol or carteolol or

beta-block* or corticosteroid* or steroid* or prednisolone or

laser* or bleomycin or lauromacrogol or therap* or treat* or

intervention*).ti,ab,kw.

5. (1 or 2) and (3 or 4)

Embase

#1 'infantile hemangioma'/exp OR

('infantile hemangioma' OR 'infantile haemangioma' OR

'hemangioma of infancy' OR 'haemangioma of infancy'):ti,ab,kw

#2 'beta adrenergic receptor blocking agent'/exp OR

'corticosteroid'/exp OR 'laser therapy'/exp OR

(propranolol OR atenolol OR nadolol OR timolol OR carteolol OR

beta-block* OR corticosteroid* OR steroid* OR prednisolone OR

laser* OR bleomycin OR lauromacrogol OR therap* OR treat* OR

intervention*):ti,ab,kw

#3 'randomized controlled trial'/exp OR 'controlled clinical trial'/exp OR

'randomization'/exp OR 'single blind procedure'/exp OR

'double blind procedure'/exp OR

(random* OR placebo* OR trial OR blind*):ti,ab

#4 #1 AND #2 AND #3
