# Supplementary material for: Efficacy and safety of interventions for infantile hemangioma compared with oral propranolol: an updated systematic review and bayesian network meta-analysis
Source: Eur J Pediatr. 2026 Jul 21;185(8):595. doi: 10.1007/s00431-026-07257-y (PMC13388786; doi:10.1007/s00431-026-07257-y)
Supplement: Supplementary file 3 — Supplementary Material 3 (DOCX 38.1 KB) [file 431_2026_7257_MOESM3_ESM.docx]

Supplementary Table S1. Detailed study characteristics

| Study_ID | Arm | Treatment (NMA Node) | Treatment Detail | Route | Dose (mg/kg/d or %) | Treatment Duration (mo) | N Randomized | N Analyzed | Events: Complete Resolution (N_clear) | Events: Adverse Events (N_ae) |
| --- | --- | --- | --- | --- | --- | --- | --- | --- | --- | --- |
| Gan LQ2018 | 1 | topical beta-blockers | carteolol | topical | NR | 3 | 224 | 224 | 38 |  |
| Gan LQ2018 | 2 | placebo | placebo | empty | NR | 3 | 125 | 125 | 7 |  |
| Christine Léauté-Labrèze2015 | 1 | propranolol | propranolol | oral | 3 | 6 | 101 | 101 | 61 | 97 |
| Christine Léauté-Labrèze2015 | 2 | placebo | placebo | oral | 3 | 6 | 55 | 55 | 2 | 42 |
| M Dakoutrou2019 | 1 | atenolol | atenolol | oral | 2 | 5 | 26 | 26 | 22 | 4 |
| M Dakoutrou2019 | 2 | propranolol | propranolol | oral | 2 | 5 | 28 | 28 | 22 | 4 |
| Alvaro Ábarzúa-Araya2014 | 1 | atenolol | atenolol | oral | 1 | 6 | 13 | 13 | 7 |  |
| Alvaro Ábarzúa-Araya2014 | 2 | propranolol | propranolol | oral | 2 | 6 | 10 | 10 | 6 |  |
| Amir Hooshang Ehsani2014 | 1 | propranolol combination | PDL with propranolol | laser and oral |  | 3 | 10 | 10 | 5 |  |
| Amir Hooshang Ehsani2014 | 2 | laser | PDL | laser |  | 3 | 9 | 9 | 2 |  |
| Hatem M Marey2017 | 1 | propranolol combination | timolol and propranolol | oral and topical | 2 | 6 | 13 | 13 | 10 |  |
| Hatem M Marey2017 | 2 | propranolol | propranolol | oral | 2 | 6 | 12 | 12 | 3 |  |
| Ashraf, Raihan2023 | 1 | atenolol | atenolol | oral | 1 | 9 | 25 | 25 | 13 |  |
| Ashraf, Raihan2023 | 2 | propranolol | propranolol | oral | 2 | 9 | 30 | 30 | 22 |  |
| Fan Ma2024 | 1 | propranolol combination | propranolol and lauromacrogol | oral and intralesional injection of lauromacrogol for 2-4 times within 6 months | 2 | 6 | 38 | 38 | 11 |  |
| Fan Ma2024 | 2 | propranolol | propranolol | oral | 2 | 6 | 29 | 29 | 11 |  |
| QY Chen2021 | 1 | topical beta-blockers | timolol | topical | 0.50% | 1 | 10 | 10 | 10 |  |
| QY Chen2021 | 2 | laser | PDL | laser |  | 1 | 9 | 9 | 9 |  |
| Yi Ji2021 | 1 | atenolol | atenolol | oral | 1 | 6 | 187 | 187 | 149 |  |
| Yi Ji2021 | 2 | propranolol | propranolol | oral | 2 | 6 | 190 | 190 | 156 |  |
| Fania Z Muñoz-Garza 2021 | 1 | topical beta-blockers | timolol | topical | 0.50% | 6 | 24 | 24 | 11 | 4 |
| Fania Z Muñoz-Garza 2021 | 2 | placebo | placebo | topical |  | 6 | 26 | 26 | 11 | 0 |
| Sunita Singh2025 | 1 | topical beta-blockers | timolol | topical | 0.50% | 6 | 20 | 20 | 18 |  |
| Sunita Singh2025 | 2 | steroid | steroid | topical | 0.05% | 6 | 20 | 20 | 20 |  |
| He Gong2015 | 1 | propranolol combination | timolol and propranolol | oral and topical | 1 | 6 | 13 | 13 | 11 |  |
| He Gong2015 | 2 | propranolol | propranolol | oral | 1.5 | 6 | 13 | 13 | 9 |  |
| He Gong2015 | 3 | topical beta-blockers | timolol | topical | 0.50% | 6 | 13 | 13 | 8 |  |
| E. Pope2013 | 1 | nadolol | nadolol | oral | 4 | 6 | 10 | 10 | 10 |  |
| E. Pope2013 | 2 | propranolol | propranolol | oral | 3 | 6 | 9 | 9 | 9 |  |
| Elena Pope2022 | 1 | nadolol | nadolol | oral | 2 | 6 | 35 | 35 | 16 | 27 |
| Elena Pope2022 | 2 | propranolol | propranolol | oral | 2 | 6 | 36 | 36 | 10 | 34 |
| Yanyan Guo2025 | 1 | propranolol | propranolol | oral | 1 | 6 | 130 | 130 | 43 | 7 |
| Yanyan Guo2025 | 2 | propranolol combination | propranolol with monthly intralesional injections of bleomycin (1 mg/mL solution) | oral | 1 | 6 | 130 | 130 | 20 | 3 |
| Hesham Zaher2025 | 1 | propranolol | propranolol | oral | 2 | 6 | 15 | 15 | 9 |  |
| Hesham Zaher2025 | 2 | topical beta-blockers | propranolol topical | topical | 1% | 6 | 15 | 15 | 3 |  |
| Hesham Zaher2025 | 3 | intralesional propranolol | 1 mg of propranolol hydrochloride in 1 ml of injection once weekly | intralesional injection | 1 | 6 | 15 | 15 | 2 |  |
| Nancy M Bauman2014 | 1 | propranolol | propranolol | oral | 2 | 4 | 11 | 11 | 9 | 1 |
| Nancy M Bauman2014 | 2 | steroid | steroid | oral | 2 | 4 | 8 | 8 | 6 | 6 |
| Preeti Tiwari2016 | 1 | propranolol | propranolol | oral | 2 | 6 | 32 | 32 | 8 | 4 |
| Preeti Tiwari2016 | 2 | placebo | placebo | oral | 10 | 6 | 32 | 32 | 3 |  |
| Xinjun Sun 2018 | 1 | propranolol combination | propranolol with Nd:YAG laser | laser and oral | 2 | 2 | 50 | 50 | 9 | 4 |
| Xinjun Sun 2018 | 2 | laser | Nd:YAG laser | laser every 3 weeks |  | 2 | 50 | 50 | 15 | 5 |
| Janneke P H M Kessels2013 | 1 | laser | PDL | laser every 4 weeks |  |  | 10 | 10 | 5 |  |
| Janneke P H M Kessels2013 | 2 | placebo | placebo | empty |  |  | 9 | 9 | 2 |  |
| Mohamed M D Aly2015 | 1 | propranolol combination | propranolol with steroid | oral | 2 | 6 | 20 | 20 | 14 | 3 |
| Mohamed M D Aly2015 | 2 | propranolol | propranolol | oral | 2 | 6 | 20 | 20 | 7 | 2 |
| Aditi Mehta2019 | 1 | intralesional propranolol | propranolol injection | intralesional injection | 1 mg/ml | 3 times | 10 | 10 | 10 |  |
| Aditi Mehta2019 | 2 | propranolol | propranolol | oral | 3 | 6 | 10 | 10 | 8 |  |
| Retno Danarti2016 | 1 | topical beta-blockers | timolol | topical | 0.5% solution | 6 | 93 | 93 | 29 |  |
| Retno Danarti2016 | 2 | steroid | steroid | topical |  | 6 | 92 | 92 | 28 |  |
| Abeer A Tawfik2015 | 1 | topical beta-blockers | timolol | topical | 0.50% | 4 | 30 | 30 | 9 |  |
| Abeer A Tawfik2015 | 2 | laser | PDL and Nd:YAG laser | laser |  | 6 | 30 | 30 | 3 |  |
| Tao Wang2024 | 1 | atenolol | atenolol | oral | 1 |  | 11 | 11 | 11 | 2 |
| Tao Wang2024 | 2 | propranolol | propranolol | oral | 2 |  | 22 | 22 | 22 | 5 |
| Shuxia Zhong2015 | 1 | propranolol | propranolol | oral | 1.5 | 6 | 20 | 20 | 6 |  |
| Shuxia Zhong2015 | 2 | laser | Nd:YAG laser | laser |  | 3 times | 20 | 20 | 2 |  |
| Shuxia Zhong2015 | 3 | propranolol combination | propranolol with Nd:YAG laser | laser and oral | 1.5 | 3 months and 3times | 20 | 20 | 17 |  |
| Wang Qi2016 | 1 | atenolol | atenolol | oral | 1 | 6 | 45 | 45 | 34 |  |
| Wang Qi2016 | 2 | propranolol | propranolol | oral | 2 | 6 | 30 | 30 | 21 |  |
| Guanjie Wang2016 | 1 | atenolol | atenolol | oral | 1 | 6 | 63 | 63 | 34 | 8 |
| Guanjie Wang2016 | 2 | propranolol | propranolol | oral | 1 | 6 | 60 | 60 | 36 | 12 |
| Longlong Sun2018 | 1 | atenolol | atenolol | oral | NR | NR | 91 | 91 | 46 | 34 |
| Longlong Sun2018 | 2 | propranolol | propranolol | oral | NR | NR | 82 | 82 | 57 | 47 |

Supplementary Table S2. Separating Indirect from Direct Evidence (SIDE) using the back-calculation method.

Panel A. Efficacy outcome (treatment success rate) — random-effects model

| Comparison | k | Prop. | NMA OR | Direct OR | Indirect OR | RoR | z | P value |
| --- | --- | --- | --- | --- | --- | --- | --- | --- |
| Intralesional propranolol vs. propranolol | 2 | 0.89 | 0.47 | 0.41 | 1.47 | 0.28 | -0.43 | 0.6638 |
| Intralesional propranolol vs. topical beta-blockers | 1 | 0.63 | 0.98 | 0.66 | 1.93 | 0.34 | -0.52 | 0.5999 |
| Laser vs. placebo | 1 | 0.28 | 2.67 | 3.00 | 2.56 | 1.17 | 0.11 | 0.9138 |
| Laser vs. propranolol | 1 | 0.24 | 0.43 | 0.30 | 0.48 | 0.63 | -0.35 | 0.7282 |
| Laser vs. propranolol combination | 3 | 0.71 | 0.31 | 0.37 | 0.20 | 1.84 | 0.51 | 0.6080 |
| Laser vs. topical beta-blockers | 1 | 0.34 | 0.89 | 0.29 | 1.60 | 0.18 | -1.30 | 0.1952 |
| Placebo vs. propranolol | 2 | 0.52 | 0.16 | 0.10 | 0.27 | 0.38 | -0.87 | 0.3824 |
| Placebo vs. topical beta-blockers | 2 | 0.62 | 0.33 | 0.50 | 0.17 | 2.85 | 0.95 | 0.3402 |
| Propranolol combination vs. propranolol | 6 | 0.89 | 1.40 | 1.89 | 0.13 | 14.47 | 2.10 | 0.0362 |
| Corticosteroids vs. propranolol | 1 | 0.35 | 0.66 | 0.68 | 0.65 | 1.05 | 0.03 | 0.9769 |
| Topical beta-blockers vs. propranolol | 2 | 0.41 | 0.48 | 0.37 | 0.57 | 0.65 | -0.41 | 0.6851 |
| Propranolol combination vs. topical beta-blockers | 1 | 0.23 | 2.90 | 2.98 | 2.88 | 1.03 | 0.02 | 0.9816 |
| Corticosteroids vs. topical beta-blockers | 2 | 0.77 | 1.38 | 1.36 | 1.43 | 0.95 | -0.03 | 0.9769 |

Panel B. Safety outcome (adverse events) — fixed-effects model

| Comparison | k | Prop. | NMA OR | Direct OR | Indirect OR | RoR | z | P value |
| --- | --- | --- | --- | --- | --- | --- | --- | --- |
| Atenolol vs. propranolol | 4 | 1.00 | 0.54 | 0.54 | — | — | — | — |
| Laser vs. propranolol combination | 1 | 1.00 | 1.28 | 1.28 | — | — | — | — |
| Nadolol vs. propranolol | 1 | 1.00 | 0.20 | 0.20 | — | — | — | — |
| Placebo vs. propranolol | 2 | 1.00 | 0.15 | 0.15 | — | — | — | — |
| Placebo vs. topical beta-blockers | 1 | 1.00 | 0.20 | 0.20 | — | — | — | — |
| Propranolol combination vs. propranolol | 2 | 1.00 | 0.66 | 0.66 | — | — | — | — |
| Corticosteroids vs. propranolol | 1 | 1.00 | 30.00 | 30.00 | — | — | — | — |

**Abbreviations:** k, number of studies providing direct evidence; Prop., proportion of direct evidence; NMA OR, estimated odds ratio from the network meta-analysis; Direct OR, odds ratio derived from direct evidence; Indirect OR, odds ratio derived from indirect evidence; RoR, ratio of ratios (direct vs. indirect); z, z-value for test of disagreement; P, P value for disagreement between direct and indirect estimates.

Statistically significant inconsistency (P < 0.05) is highlighted in yellow with bold text. In Panel B, all comparisons with direct evidence had a direct evidence proportion of 1.00, meaning no indirect evidence was available for comparison; therefore, inconsistency testing was not applicable ("—").

Supplementary Table S3. Frequentist network meta-analysis results (netmeta validation).

| Prop. comb. | 1.53 (0.20–11.83) | 0.72 (0.32–1.58) | 2.11 (0.41–10.93) | 0.55 (0.19–1.60) | 0.34 (0.05–2.42) | 2.90 (0.92–9.17) | 0.31 (0.11–0.89) | 0.11 (0.03–0.39) |
| --- | --- | --- | --- | --- | --- | --- | --- | --- |
| 0.30 (0.04–2.18) | Nadolol | 2.14 (0.32–14.09) | 3.22 (0.28–36.47) | 0.36 (0.05–2.71) | 0.22 (0.02–3.07) | 4.44 (0.52–37.85) | 0.20 (0.02–1.81) | 13.31 (1.52–116.81) |
| 1.52 (0.50–4.65) | 0.20 (0.04–1.01) | Propranolol | 1.46 (0.11–19.41) | 0.77 (0.38–1.57) | 0.47 (0.08–2.96) | 2.67 (0.55–13.04) | 0.43 (0.14–1.32) | 0.16 (0.05–0.47) |
| 0.02 (0.00–0.37) | 0.01 (0.00–0.14) | 0.03 (0.00–0.45) | Cortico- steroids | 1.16 (0.21–6.26) | 0.71 (0.07–6.92) | 1.38 (0.35–5.36) | 0.65 (0.11–3.68) | 0.24 (0.05–1.24) |
| 0.82 (0.24–2.74) | 2.70 (0.49–14.73) | 0.54 (0.33–0.86) | 0.02 (0.00–0.25) | Atenolol | 1.63 (0.23–11.73) | 1.60 (0.46–5.55) | 1.79 (0.47–6.80) | 4.80 (1.32–17.47) |
| — | — | — | — | — | IL prop. | 0.98 (0.14–6.70) | 1.10 (0.14–8.88) | 2.94 (0.38–22.55) |
| 0.88 (0.06–13.56) | 0.27 (0.01–5.25) | 1.34 (0.11–16.28) | 40.21 (1.09–1484.33) | 0.72 (0.06–9.12) | — | Topical BB | 0.89 (0.26–3.06) | 0.33 (0.12–0.95) |
| 1.28 (0.32–5.07) | 4.23 (0.38–46.96) | 0.84 (0.14–4.94) | 0.03 (0.00–0.65) | 0.64 (0.10–4.00) | — | 1.12 (0.05–24.04) | Laser | 2.67 (0.73–9.83) |
| 0.23 (0.05–1.05) | 1.33 (0.19–9.21) | 0.15 (0.05–0.42) | 0.00 (0.00–0.08) | 3.59 (1.14–11.29) | — | 0.20 (0.02–1.93) | 5.62 (0.72–43.98) | Placebo |

Upper triangle (read left to right): efficacy (treatment success rate), expressed as OR (95% CI); an OR > 1 favors the row treatment over the column treatment. Lower triangle (read left to right): safety (adverse events), expressed as OR (95% CI); an OR > 1 indicates a higher likelihood of adverse events for the column treatment compared with the row treatment. Statistically significant results (95% CI excluding 1) are shown in bold with green shading. This table presents frequentist estimates from the netmeta package as a sensitivity analysis to validate the primary Bayesian results (Table 2). OR, odds ratio; CI, confidence interval; IL prop., intralesional propranolol; Prop. comb., propranolol combination therapy; Topical BB, topical beta-blockers; Cortico., corticosteroids.

Supplementary Table S4: Certainty of evidence assessment using CINeMA for the efficacy outcome.

| Comparison | Number of studies | Within-study bias | Reporting bias | Indirectness | Imprecision | Heterogeneity | Incoherence | Confidence rating | Reason(s) for downgrading |
| --- | --- | --- | --- | --- | --- | --- | --- | --- | --- |
| atenolol:propranolol | 7 | Some concerns | Low risk | No concerns | Some concerns | Some concerns | Some concerns | Low | ["Within-study bias","Imprecision","Heterogeneity","Incoherence"] |
| intralesional propranolol:propranolol | 2 | Major concerns | Low risk | No concerns | Major concerns | No concerns | No concerns | Very low | ["Within-study bias","Imprecision"] |
| laser:propranolol | 1 | Major concerns | Low risk | No concerns | Some concerns | Some concerns | No concerns | Very low | ["Within-study bias","Imprecision","Heterogeneity"] |
| nadolol:propranolol | 1 | No concerns | Low risk | No concerns | Major concerns | No concerns | Some concerns | Very low | ["Imprecision","Incoherence"] |
| placebo:propranolol | 2 | Some concerns | Low risk | No concerns | No concerns | Some concerns | No concerns | Moderate | ["Within-study bias","Heterogeneity"] |
| propranolol:propranolol combination | 6 | Major concerns | Low risk | No concerns | Some concerns | Some concerns | Major concerns | Very low | ["Within-study bias","Imprecision","Heterogeneity","Incoherence"] |
| propranolol:steroid | 1 | Some concerns | Low risk | No concerns | Major concerns | No concerns | No concerns | Very low | ["Within-study bias","Imprecision"] |
| propranolol:topical beta-blockers | 2 | Major concerns | Low risk | No concerns | Some concerns | Some concerns | No concerns | Very low | ["Within-study bias","Imprecision","Heterogeneity"] |

Supplementary Table S5. Sensitivity analysis league table for efficacy outcome (excluding high risk-of-bias studies).

|  | Propranolol combination | Nadolol | Propranolol | Atenolol | Steroid | Topical beta-blockers | Placebo |
| --- | --- | --- | --- | --- | --- | --- | --- |
| Prop. comb. | Prop. comb. | 0.18 (0.00–7.13) | 0.08 (0.00–1.33) | 0.06 (0.00–1.13) | 0.01 (0.00–0.92) | 0.01 (0.00–0.26) | 0.00 (0.00–0.11) |
| Nadolol | 5.54 (0.14–251.64) | Nadolol | 0.45 (0.04–5.06) | 0.32 (0.02–4.48) | 0.06 (0.00–4.03) | 0.03 (0.00–1.11) | 0.02 (0.00–0.44) |
| Propranolol | 12.29 (0.75–244.32) | 2.22 (0.20–25.56) | Propranolol | 0.71 (0.19–2.27) | 0.14 (0.02–3.24) | 0.07 (0.01–0.96) | 0.04 (0.00–0.34) |
| Atenolol | 17.52 (0.88–468.64) | 3.12 (0.22–51.98) | 1.41 (0.44–5.18) | Atenolol | 0.20 (0.02–6.64) | 0.10 (0.01–1.91) | 0.06 (0.00–0.70) |
| Steroid | 88.26 (1.09–2892.34) | 16.14 (0.25–308.02) | 7.06 (0.31–64.74) | 5.03 (0.15–54.76) | Steroid | 0.56 (0.04–2.01) | 0.28 (0.01–2.01) |
| Topical BB | 169.65 (3.78–8658.29) | 30.22 (0.90–1084.41) | 13.55 (1.04–188.12) | 9.59 (0.52–165.78) | 1.79 (0.50–26.90) | Topical BB | 0.52 (0.06–4.35) |
| Placebo | 324.03 (9.32–13900.08) | 56.62 (2.27–1706.97) | 25.54 (2.93–274.74) | 18.14 (1.42–248.01) | 3.53 (0.50–96.21) | 1.91 (0.23–17.43) | Placebo |

Sensitivity analysis restricted to 13 studies at low risk of bias or with some concerns (random-effects consistency model). Treatments on the diagonal are ordered by SUCRA ranking for efficacy (highest to lowest). Each cell presents the odds ratio (95% credible interval); an OR > 1 favors the column treatment over the row treatment. Statistically significant results (95% CrI excluding 1) are shown in bold with green shading. Laser therapy and intralesional propranolol were excluded from this analysis as all contributing trials were classified as high risk of bias. OR, odds ratio; CrI, credible interval; SUCRA, surface under the cumulative ranking curve; Prop. comb., propranolol combination therapy; Topical BB, topical beta-blockers.
