# Supplementary material for: Efficacy and safety of interventions for infantile hemangioma compared with oral propranolol: an updated systematic review and bayesian network meta-analysis
Source: Eur J Pediatr. 2026 Jul 21;185(8):595. doi: 10.1007/s00431-026-07257-y (PMC13388786; doi:10.1007/s00431-026-07257-y)
Supplement: Supplementary file 1 — Supplementary Material 1 (DOCX 13.3 MB) [file 431_2026_7257_MOESM1_ESM.docx]

Supplementary Figure S1: Risk of Bias Traffic Light Plot

Supplementary Figure S1 Legend: Study identification numbers corresponding to the Risk of Bias traffic light plot.

| **#** | **Study** | **#** | **Study** | **#** | **Study** |
| --- | --- | --- | --- | --- | --- |
| 1 | Gan 2018 | 11 | Muñoz-Garza 2021 | 21 | Kessels 2013 |
| 2 | Léauté-Labrèze 2015 | 12 | Singh 2025 | 22 | Aly 2015 |
| 3 | Dakoutrou 2019 | 13 | Gong 2015 | 23 | Mehta 2019 |
| 4 | Ábarzúa-Araya 2014 | 14 | Pope 2013 | 24 | Danarti 2016 |
| 5 | Ehsani 2014 | 15 | Pope 2022 | 25 | Tawfik 2015 |
| 6 | Marey 2017 | 16 | Guo 2025 | 26 | Wang T 2024 |
| 7 | Ashraf 2023 | 17 | Zaher 2025 | 27 | Zhong 2015 |
| 8 | Ma 2024 | 18 | Bauman 2014 | 28 | Wang Q 2016 |
| 9 | Chen 2021 | 19 | Tiwari 2016 | 29 | Wang G 2016 |
| 10 | Ji 2021 | 20 | Sun X 2018 | 30 | Sun L 2018 |

Study numbering corresponds to the order presented in Table 1.

Supplementary Figure S2: Risk of Bias Summary Plot

Risk of bias summary of included studies. Bar plot showing the proportion of studies with low risk (green), some concerns (yellow), or high risk (red) of bias across the five domains of the RoB 2 tool.

Supplementary Figure S3: Comparison-Adjusted Funnel Plots

A

B

B

A

**Supplementary Figure 4.** Cumulative ranking probability plots (rankograms) for efficacy (**A**) and safety (**B**) outcomes. The y-axis represents the cumulative probability of each treatment achieving a given rank or better. Higher curves indicate more favorable rankings. SUCRA, Surface Under the Cumulative Ranking Curve.
